# Intraspecific genotypic variability determines concentrations of key truffle volatiles

**DOI:** 10.1111/j.1469-8137.2012.04077.x

**Published:** 2012-05

**Authors:** Richard Splivallo, Nayuf Valdez, Nina Kirchhoff, Marta Castiella Ona, Jean-Pierre Schmidt, Ivo Feussner, Petr Karlovsky

**Affiliations:** 1 Molecular Phytopathology and Mycotoxin Research, University of GoettingenGrisebachstrasse 6, D-37077 Goettingen, Germany; 2 41A routes des AnnuairesCH-1870, Switzerland; 3 Department of Plant Biochemistry, Albrecht von Haller Institute for Plant Sciences, Georg August UniversityJustus-von-Liebig Weg 11, D-37077 Göttingen, Germany

**Keywords:** 1-octen-3-ol, amplified fragment length polymorphism (AFLP), aroma variability, truffle genetics, *Tuber borchii*, *Tuber* spp., *Tuber uncinatum*, volatile organic compounds (VOCs)

## Abstract

- Aroma variability in truffles has been attributed to maturation (*Tuber borchii*), linked to environmental factors (*Tuber magnatum*), but the involvement of genetic factors has been ignored. We investigated aroma variability in *Tuber uncinatum*, a species with wide distribution. Our aim was to assess aroma variability at different spatial scales (i.e. trees, countries) and to quantify how aroma was affected by genotype, fruiting body maturity, and geographical origin.
- A volatile fingerprinting method was used to analyze the aroma of 223 *T. uncinatum* fruiting bodies from seven European countries. Maturity was estimated from spore melanization. Genotypic fingerprinting was performed by amplified fragment length polymorphism (AFLP).
- Discriminant analysis revealed that, regardless of the geographical origin of the truffles, most of the aroma variability was caused by eight-carbon-containing volatiles (C8-VOCs). In an orchard of *T. uncinatum*, truffles producing different concentrations of C8-VOCs clustered around distinct host trees. This clustering was not associated with maturity, but was associated with fungal genotype.
- These results indicate that the variation in C8-VOCs in truffles is most likely under genetic control. They exemplify that understanding the factors behind aroma variability requires a holistic approach. Furthermore, they also raise new questions regarding the ecological role of 1-octen-3-ol in truffles.

## Introduction

Like plants and bacteria, fungi synthesize an enormous diversity of volatile organic compounds (VOCs) which might be involved in communication with above- and belowground organisms (Wenke *et al.*, 2010). Variability in the volatile constituents within single fungal species has been documented many times; neither its causes nor its implications for the fitness of its producers are well understood.

Hundreds of VOCs have been described from the fruiting bodies of truffles (Diaz *et al.*, 2003; Mauriello *et al.*, 2004; March *et al.*, 2006; Gioacchini *et al.*, 2008; Culleré*et al.*, 2010; Splivallo *et al.*, 2011); however, only a few have been implicated in communication with other organisms (Splivallo *et al.*, 2011). These include dimethyl sulfide (DMS), which acts as an attractant to dogs and pigs (Talou *et al.*, 1990), and eight-carbon-containing volatiles (C8-VOCs), predominantly 1-octen-3-ol, that might act as signals to plants (Splivallo *et al.*, 2007b, 2011). The biosynthesis of these volatiles in truffles has not been fully elucidated but might involve amino acid catabolism (for generation of small sulfur-VOCs; Martin *et al.*, 2010; Splivallo *et al.*, 2011), or fatty acid degradation (i.e. for generation of 1-octen-3-ol from linoleic acid, as occurs in other fungi; Wurzenberger & Grosch, 1984; Brodhun *et al.*, 2009). These VOCs might also result from a complex interplay between the fungus and associated microbes (Buzzini *et al.*, 2005; Splivallo *et al.*, 2011).

A typical volatile profile of a single truffle fruiting body contains 20–50 VOCs (Splivallo *et al.*, 2007a; Culleré*et al.*, 2010). The aroma does qualitatively and quantitatively vary among fruiting bodies within the same truffle species (Mauriello *et al.*, 2004; Zeppa *et al.*, 2004; Gioacchini *et al.*, 2008; Splivallo *et al.*, 2011). Variability in fungal aroma can be attributed to many factors, including genotypic variability, differences in developmental stage or maturation, and the influence of external abiotic and biotic factors.

Genotypic variability among strains of the wine yeast *Saccharomyces cerevisiae* affects the quality and quantity of wine aromas (Mendes-Ferreira *et al.*, 2010; Borneman *et al.*, 2011). Estimating the extent to which genotypic variability shapes fungal aroma requires an understanding of all biosynthetic pathways involved in volatile synthesis, which has not yet been achieved for any fungus.

Fungal development (hyphal aggregation) is another factor that shapes intraspecific volatile variability. In the mushrooms *Agaricus bisporus* and *Tricholoma matsutake*, the cap has a distinct smell from the stipe because of differences in volatile composition of both pseudotissues (Wurzenberger & Grosch, 1983; Cho *et al.*, 2008).

Aging and maturation can further influence fungal aroma, as has been demonstrated for the straw mushroom *Volvariella volvacea*, in which the concentrations of two volatiles with characteristic fungal odor, 1-octen-3-ol and 3-octanone, change during maturation (Zhang *et al.*, 2008).

Abiotic factors such as temperature or media composition also affect fungal volatile profiles (Molina *et al.*, 2007). Media composition, for example, qualitatively influences the volatile profiles of truffle mycelia (Splivallo *et al.*, 2007a; Tang *et al.*, 2009). With respect to biotic factors, many studies have documented that pathogens and herbivores influence volatile emission from plants (Karban & Baldwin, 1997; Leitner *et al.*, 2005) but much less is known about how biotic factors affect volatile emission from fungi. The preference of collembola for ungrazed mycelium, however, suggests that fungi release specific volatile cues upon grazing (Staaden *et al.*, 2011).

What causes intraspecific aroma variability in truffles and other fungi is not well understood (Splivallo *et al.*, 2011). In white truffles, fruiting body maturation and geographical origin have been reported to qualitatively influence aroma (Zeppa *et al.*, 2004; Gioacchini *et al.*, 2008). Because their study of the black truffle *Tuber melanopsorum* detected no population structure based on random amplification of polymorphic DNA (RAPD) and microsatellites, Bertault *et al.*, 1998 argued that intraspecific variability in truffle aroma should be attributed to environmental factors. In more recent studies, however, a low degree of population structure and polymorphism was detected in *T. melanopsorum* using single nucleotide polymorphism and other microsatellites (Murat *et al.*, 2004, 2011). Genetic diversity has also been documented in other truffle species, including *T. uncinatum* and its *Tuber aestivum* morphotype, the species used in the current study (Mello *et al.*, 2002, 2005; Paolocci *et al.*, 2004). Despite the documented genetic diversity within single truffle species, the argument by Bertault *et al.* (1998) is still repeated in papers that focus either on aroma variability or on genotypic variability in truffles but do not consider both factors together (Bertault *et al.*, 1998; Paolocci *et al.*, 2004; Gioacchini *et al.*, 2008).

One aim of the current study concerned intraspecific variability in truffle aroma at different spatial scales. We focused on *T. uncinatum* instead of *Tuber melanosporum*, the species whose genome has been recently sequenced (Martin *et al.*, 2010; Kües & Martin, 2011), because *T. uncinatum* is distributed throughout Europe, whereas *T. melanosporum* is mostly confined to France, Italy, and Spain. This wide geographical distribution makes *T. uncinatum* an excellent candidate for studying aroma variability on a large geographical scale.

Our aims here were twofold. As noted previously, our first aim was to assess aroma variability within *T. uncinatum* at different scales (among countries; within the same country; within truffles collected from different host plants within the same truffle orchard). Our second aim was to quantify how aroma variability within *T. uncinatum* is affected by genotypic variability, fruiting body maturity, and geographical origin.

## Materials and Methods

### Truffle fruiting bodies and sampling strategy

**Fig. 1:**
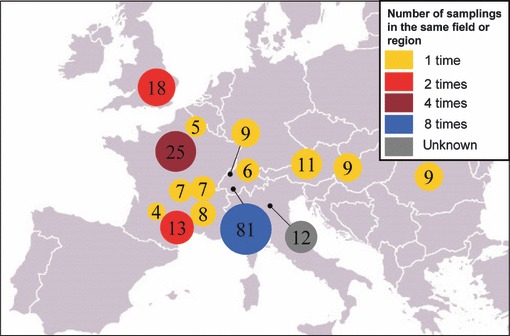
Sampling of *Tuber uncinatum* fruiting bodies. A total of 223 *T. uncinatum* fruiting bodies were collected between 2008 and 2011 from seven European countries. The numbers in the circles indicate the number of fruiting bodies collected from a single site, and the color coding indicates the number of times a single region was sampled. Further details about sampling sites and sampling dates are provided in Supporting Information Table S1.

A total of 223 fruiting bodies of *Tuber uncinatum* (morphotypes *T. uncinatum* Chatin and *T.* *aestivum* Vittad.) (Paolocci *et al.*, 2004) were collected over 4 yr (2008–2011) from seven European countries including France, Italy, Switzerland, the UK, Austria, Romania, and Hungary (Fig. 1). Unless mentioned otherwise, samples were stored at 4°C immediately after collection and frozen at −70°C within 6 d. Seasonal variability in the aroma of fruiting bodies was assessed by repeatedly sampling some sites over time (between two and eight times; Fig. 1). Furthermore, mapping the distribution of fruiting bodies/volatile compounds within a single field was achieved by recording the exact location of fruiting bodies in an artificially inoculated field in Wallis, Switzerland. All truffle samples collected from the field in Wallis, Switzerland, were frozen to −70°C within 3 h of collection to prevent any bias in volatile profiles as a result of extended sample storage at 4°C.

The sampling date, number of specimens collected per site, host tree, and field type (artificial or natural truffle orchards) are listed in the Supporting Information, Table S1. To comply with the requests of the truffle hunters who provided us with biological material to maintain the secrecy of the location of the collection sites, we have only listed in Table S1 the closest city to the collection site. Identification of *T. uncinatum* was based on the morphology of mature/ornamented spores from ascii and by PCR using the *T. uncinatum*-specific primers UNCI/UNCII for fully immature fruiting bodies (Mello *et al.*, 2002).

Maturity was determined for each fruiting body by calculating the ratio between the number of ascii containing melanized spores and the total number of ascii (Zeppa *et al.*, 2004). Samples were categorized according to Zeppa *et al.* (2004) into four maturity groups according to the percentage of ascii containing mature spores (stage 0, 0–5%; stage 1, 6–30%; stage 2, 31–70%; stage 3, 71–100%).

Fruiting bodies of *Tuber borchii*, *Tuber brumale*, *Tuber macrosporum*, *Tuber indicum* and *Tuber magnatum* were purchased from wholesalers in France, Italy, Germany, and Hungary between 2008 and 2010. Species confirmation was based on the morphology of mature/ornamented spores.

### Amplified fragment length polymorphism (AFLP) analysis

DNA was extracted from pieces of gleba (*c.* 100 mg per sample) removed from the inner part of the *T. uncinatum* fruiting bodies. The DNA was extracted with the InnuPREP plant DNA kit (Analytik Jena AG, Jena, Germany), and DNA samples were further purified with ethidium bromide/high-salt buffer (Stemmer, 1991).

Amplified fragment length polymorphism analysis of *T. uncinatum* DNA was performed according to Vos *et al.* (1995), except that *Tru*1I replaced *Mse*I, using adapters described before (Reineke & Karlovsky, 2000). In brief, DNA (100 ng per sample) was restricted with the enzymes *Eco*RI and *Tru*1I (Fermentas GmbH, St. Leon-Rot, Germany) in a 10 μl reaction volume. Adapter molecules were attached in a 20 μl reaction volume containing 10 μl of double-restricted DNA, 5 pmol Eco-Adapter 1, 5 pmol Eco-Adapter 2, 50 pmol *Tru*1I-Adapter 1, 50 pmol *Tru*1I-Adapter 2, T4-DNA-ligase-buffer (Fermentas), and 0.25 U of T4-DNA-Ligase. Preamplification with unlabeled primers was performed as described previously (Reineke & Karlovsky, 2000). The products were diluted 1 : 10 in double-distilled water, and 1 μl was used as template for the amplification using primers with selective nucleotides. Each reaction contained 5 pmol labeled *Eco*RI primer, 10 pmol *Tru*II-primer, PCR buffer (Bioline GmbH, Luckenwalde, Germany), 3 mM MgCl_2_, 0.2 mM dNTPs (Fermentas), 1.5 U of Taq-DNA-polymerase (Bioline), and double-distilled water to a total volume of 25 μl. The following labelled primers were purchased from Purimex GmbH (Germany): *Eco*RI-ACA_Dy635 (5′-GACTGCGTACCAATTCACA-3′), *Eco*RI-AGC_Dy680 (5′-GACTGCGTACCAATTCAGC-3′) and *Eco*RI-ACC_Dy750 (5′-GACTGCGTACCAATTCACC-3′). Primers *Tru*II-CAC (5′-CATGAGTCCTGAGTAACAC-3′) and *Tru*II-CAG (5′-CATGAGTCCTGAGTAACAG-3′) were purchased from Invitrogen. Four different primer pairs were used for the selective PCR: *Tru*II-CAC and *Eco*RI-ACA_Dy635; *Tru*II-CAC and *Eco*RI-AGC Dy680; *Tru*II-CAG and *Eco*RI-ACA_Dy635; *Tru*II-CAG and *Eco*RI-ACC_Dy750. The selective PCR reaction was performed using a touchdown protocol (Reineke & Karlovsky, 2000).

PCR products were separated by capillary electrophoresis (CEQ8000, Beckman Coulter GmbH, Krefeld, Germany) under the following conditions: denaturation at 90°C for 120 s and separation for 70 min by 4.8 kV.

The AFLP data were analyzed with the CEQ™ software from Beckmann Coulter GmbH. Fragment recognition was performed with the following parameters: a maximum bin-width of three nucleotides, a slope threshold of 15%, and a relative peak height threshold of 10%. The confidence level was set at 95%. The applied model for calibration was the quartic curve model, which is recommended by Beckman when the Standard 600 is used. The output was transformed into a presence/absence matrix. The matrix was then analyzed with the programs NTSYS 2.0 (for clustering) and Winboot (for calculating the bootstrap values after 2000 bootstrap-replications). The dendrogram was constructed using Jaccard’s similarity coefficients and UPGMA.

### Fruiting bodies preparation for volatile analysis

Samples stored at −70°C were removed from the freezer and allowed to equilibrate on ice to −10 to −5°C. A small piece of gleba (300–400 mg) was immediately excised with a cork borer from the central part of the fruiting bodies and transferred to a 20 ml solid-phase microextraction (SPME) vial equipped with a screw cap and a silicon/polytetrafluoroethylene (PTFE) septum (La-Pha-Pack® GmbH, Langerwehe, Germany). Before sample preparation, all SPME vials were washed, rinsed with distilled water, and dried at 160°C for 3 h to minimize contamination.

### Volatile extraction by SPME

Volatiles were extracted by SPME, a semiquantitative method that allows the quick processing of a large number of samples compared with classical quantitative analytical approaches (Risticevic *et al.*, 2010). Extraction was performed at 25 ± 1°C. We compared four SPME fibers (PDMS, PA, PDMS/CARB and PDMS/DVB) and three extraction times (5, 10, and 15 min) to optimize signal intensity. The PDMS/DVB fiber was selected for analysis of all samples because of its highest extraction efficiency (an exposure time of 10 min resulted in equilibrium extraction). Empty vials were included as controls. Before each analysis, the SPME fiber was conditioned according to the supplier’s instructions. Blank runs were regularly performed between samples to ensure that no carryover occurred during chromatography.

### SPME-GC-MS volatile fingerprinting

The volatile profiles of all truffles collected between 2008 and 2011 were analyzed on an ongoing basis (after a maximum of 3 months at −70°C) to avoid any bias as a result of extended sample storage.

An Agilent GC (model 6890GC, Agilent Technologies, Deutschland GmbH, Böblingen, Germany) equipped with a quadrupole mass detector (model 5973 Network Mass detector) was used to generate volatile profiles. Volatiles were separated with a capillary column HP-5MS Agilent 19091S-433 (0.25 mm × 30 m × 0.25 μm) using the following 6.46 min temperature program: start at 60°C and hold for 2 min; ramp at 15°C min^−1^ to 80°C; ramp at 80°C min^−1^ to 250°C; hold for 1 min. Helium was used as a carrier gas with a flow rate of 1 ml min^−1^. MS parameters were set to a mass-to-charge ratio (*m*/*z*) scan range from 50 to 450 with the quadrupole detector in autotune mode, ion M+, operating at 70 eV (MS source 150°C, MS quad 230°C).

### Data processing for SPME-GC-MS fingerprints of volatiles

Three-dimensional data consisting of *m*/*z* values, scan numbers (equivalent to retention times), and signal intensities were obtained from each sample through MSD ChemStation (D01.01.16, Agilent Technologies). To account for changes in retention as a result of machine drift, we realigned peaks through an in-house developed Perl script. The data after alignment consisted of a two-dimensional matrix in which the column headers indicated the sample names (for the sample codes, refer to Table S1), row headers indicated the signals represented by a specific *m*/*z* and retention window, and values were the *m*/*z* intensities (Table S2). Signals present in the empty control vials were subtracted from each gleba sample before further analysis.

Because differences in sample biomass had a negligible effect on SPME-GC-MS signal intensity (Fig. S2), the data were not normalized to sample biomass; nor was an internal standard used for SPME-GC-MS because our preliminary results (not shown) indicated that the variability in volatile profiles among the samples was much larger than any possible matrix effects.

**Fig. 2:**
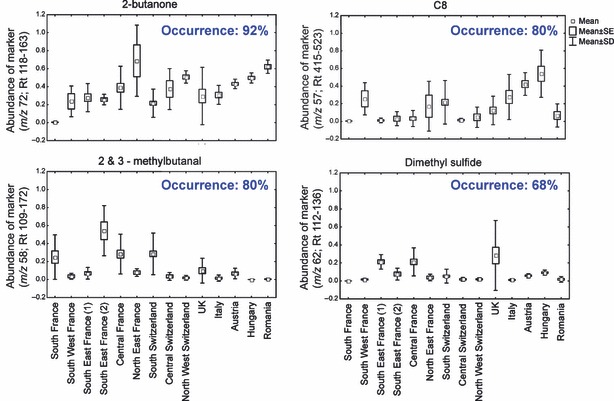
Distribution of major volatiles of *Tuber uncinatum* among geographical regions. A volatile marker was defined as major when it was detected in > 65% of all samples listed in Supporting Information Table S2. Abundance refers to the normalized intensity (values in Table S2 divided by the maximum intensity of a given marker). The four major volatile markers were associated with the presence of 2-butanone, C8 volatiles (1-octen-3-ol, 3-octanone, 3-octanol, 2-octen-1-ol, and *trans*-2-octenal), 2-methylbutanal, 3-methylbutanal, and dimethyl sulfide in the samples.

**Fig. 3:**
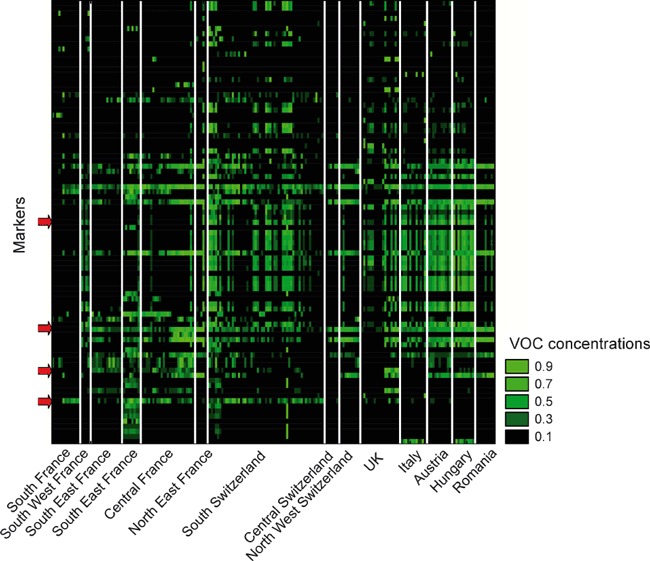
*Tuber uncinatum* truffles collected from different regions/seasons can be distinguished based on fingerprints of their volatile organic compounds (VOCs). The heat map was generated from data in Supporting Information Table S2 by selecting volatile markers that significantly differed among collection sites (*P* < 0.01; Kruskal–Wallis test). Values for all markers are normalized: light green indicates high concentrations while black indicates low concentrations. Distinct patterns are evident for some sites (different sites/regions are separated by a white rectangle). The variability associated with most VOC markers within a single site was substantial, as illustrated by the red arrows which correspond, from top down, to the following markers: C8-VOCs (*m*/*z* 57; Rt415-523); 2-butanone (*m*/*z* 72; Rt118-163); dimethyl sulfide (*m*/*z* 62; Rt112-136) and 2-methylbutanal and 3-methylbutanal (*m*/*z* 58; Rt109-172).

**Fig. 4:**
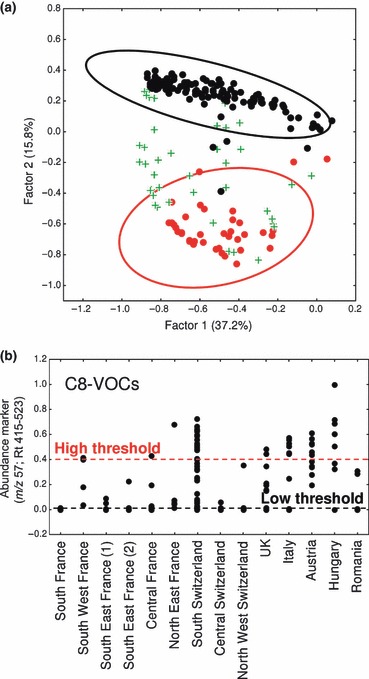
C8 volatiles (C8-VOCs) explain a substantial percentage of the variability in VOC profiles of *Tuber uncinatum.* (a) Scatterplot illustrating factor loadings (principal factor analysis) with 95% confidence ellipses generated from the data in Supporting Information Table S2 (after data normalization). Samples were grouped in three categories based on marker (*m*/*z* 57; Rt 415-523), reflecting the occurrence of C8-VOCs (high (red), > 0.40; medium (green), > 0.04; low (black), ≥ 0.00). (b) Normalized intensities of marker (*m*/*z* 57; Rt 415-523) for all samples grouped by geographical origin indicate that fruiting bodies producing high and low concentrations of C8-VOCs are in many cases present in the same geographical location/truffle field.

Throughout the manuscript the term ‘normalization’ or ‘normalized’ indicates that the abundance of one volatile marker has been divided by its maximum abundance from Table S2. This ‘normalization’ was performed to be able to compare the variability among the four major volatile markers of Fig. 2, in order to visualize the heatmap of Fig. 3 properly and to avoid giving an excessive weight to C8 markers in the principal factor analysis of Fig. 4.

### Sample homogeneity in VOC profiles and data reproducibility

The variability in VOC profiles within single fruiting bodies was assessed by analyzing three to four pieces from each of seven *T. uncinatum* truffles of various geographical origins. The standard error for selected VOC markers was < 10%, indicating high homogeneity of VOC distribution within single fruiting bodies. Because the 10% variability within a single fruiting body was much smaller than the variability among different fruiting bodies in our data set, a single replicate per sample was considered sufficient to measure the variability in VOC profiles.

### Effect of sample mass on SPME-GC-MS volatile fingerprints

The weight of all gleba samples used for volatile fingerprinting by SPME-GC-MS was 300–400 mg. To assess the effect of the sample mass on the volatile fingerprint, one large *T. uncinatum* truffle was subdivided in 3 × (300 ± 5 mg) and 3 × (400 ± 5 mg). Volatile fingerprints were generated for these samples by SPME-GC-MS.

### Effect of sample storage at 4°C on SPME-GC-MS volatile fingerprints

Since only the truffle samples collected from the field in Wallis, Switzerland, were frozen immediately after collection, and other truffles were stored at 4°C up to 6 d, we checked whether storage at 4°C would induce large changes in volatile profiles. For this purpose nine *T. uncinatum* truffles (producing varying quantities of C8-VOCs) were divided in three pieces; the first piece was frozen immediately after collection, and the second and third pieces were frozen after 3 and 6 d of storage at 4°C, respectively. Volatile fingerprints were generated for the nine samples (400 ± 5 mg) by SPME-GC-MS and the data were processed as explained earlier.

### Identification of volatile markers (maturity or site specific)

Volatile signals (*m*/*z*; retention time window) were considered to be markers for samples from a given collection site/time or of a given maturity category if they differed significantly from signals from other collection sites/times or maturity categories (*P* < 0.01, Kruskal–Wallis test performed in R; Hollander & Wolfe, 1973).

Principal factor analysis (performed in Statistica, 9.0, StatSoft GmbH, Hamburg, Germany) was used to visualize volatile profiles in two-dimensional scatterplots; the method was preferred to principal component analysis because of its superior ability to detect structure in data (Costello & Osborne, 2005).

### Calculation of diversity indices based on the SPME-GC-MS fingerprints

Volatile similarity indices were calculated for pairs of sites or collection times based on Table S2. By analogy to Nei’s genetic identity (Nei, 1972, 1978), the similarity index was defined as *I*_VOC_ = *V*_AB_/(*V*_A_ × *V*_B_)^1/2^, where *V*_A_ is the sum of the frequencies squared of all volatile markers in site A, *V*_B_ is the sum of the frequencies squared of all volatile markers in site B, and *V*_AB_ is the sum of the product of the frequencies of volatile markers occurring at either sites A or B. The frequency of a single volatile marker for one site/time point is equal to the ratio between the numbers of fruiting bodies in which the volatile could be detected and the total number of fruiting bodies collected at that site/time point.

### Structure assignments of volatile markers (SPME-GC-MS)

Volatiles structures were identified based on comparison of fragmentation patterns to the National Institute of Standards and Technology (NIST) library (version 2.0f, 25 June 2008) as well as on Kovats indices. The identities of 2-butanone, DMS, 2-methylbutanal, 3-methylbutanal, and 1-octen-3-ol were confirmed with authentic standards (purchased from Sigma-Aldrich).

### Quantification of C8-VOCs in *T. uncinatum* fruiting bodies by open loop stripping-gas chromatography-flame ionization detection (OLS-GC-FID)

Because SPME-GC-MS was used in our study as a semiquantitative method (no internal standard), we quantified C8-VOCs (1-octen-3-ol, 3-octanone, 3-octanol, and trans-2-octenal) from a subset of samples with a different quantitative analytical approach (using an internal standard). C8-VOCs were quantified from gleba samples (400 ± 5mg) by OLS-GC-FID in 34 samples (27 samples from Switzerland, three from France, and four from Italy) as follows. Each gleba sample was placed in a 100 ml Erlenmeyer flask sealed with a silicone stopper containing a stainless steel inlet and outlet, and flushed for 3 h with air (0.8 ml min^−1^) previously purified on a charcoal filter. An activated charcoal cartridge (5 mg) was placed directly at the outlet of flask and served as a trap for the volatiles emitted by the fruiting body. After volatile collections, the traps were flushed with 100 μl dichloromethane containing 1 μg nonyl acetate as internal standard. The samples (1 μl) were injected on a GC-FID (2 : 1 split). Volatiles were separated on a capillary column HP-5 Agilent 19091J-413 (0.32 mm × 30 m × 0.25 μm) using the following 60 min temperature program: start at 40°C and hold for 2.67 min; ramp at 2°C min^−1^ to 140°C and hold for 2 min; ramp at 50°C min^−1^ to 250°C; hold for 3.13 min. Helium was used as a carrier gas with a flow rate of 1 ml min^−1^. C8-VOCs were identified by injecting commercial C8 standards and quantified using the internal standard on the FID.

### Volatiles produced by mycelial cultures of truffles

Two-month-old mycelial cultures of *T. borchii* (strains ATCC 96540 and 43BO provided by Prof. A. Zambonelli, University of Bologna, Italy) and *T. melanosporum* strain TN1 (provided by Dr G. Chevalier, INRA Clermont-Ferrand, France) grown on malt extract agar (1%, Difco GmbH, Augsburg, Germany; pH adjusted to 7.0) were transferred (1.0 colony for *T. borchii* and 2.5 for *T. melanosporum*) to 250 ml Erlenmeyer flasks containing 80 ml of malt extract broth (1%, pH 7.0). The cultures were homogenized with a sterile blender, and kept in the dark at 23°C for 2 months. Fully grown cultures were homogenized again with a sterile blender, transferred to 50 ml Falcon tubes, and pelleted by centrifugation at 2700 ***g*** for 10 min. A 2.0 g quantity of the mycelial pellet (or culture broth as a control) was transferred to a 20 ml (SPME) vial for volatile analysis following the protocol used for fruiting bodies. This experiment was performed with four independent biological replicates per truffle strain.

## Results

### Effect of chromatographic resolution, sample mass, sample storage and matrix effect on volatile fingerprints

Fingerprints of volatiles were generated by SPME-GC-MS for 223 fruiting bodies of *T. uncinatum* collected from seven geographical regions (Fig. 1, Table S1).

The fingerprints for all samples, consisting of volatile markers defined by their *m*/*z* ratios and retention time windows, are displayed in Table S2. In obtaining the fingerprints with chromatography, we used a short run time of 6.46 min rather than 60 min, which is typically used for truffles (Splivallo *et al.*, 2007a; Gioacchini *et al.*, 2008), so that we could quickly process numerous samples. The chromatograms (total ion current of the SPME-GC-MS) of three truffles originating from Switzerland, France, and Italy (Fig. S1) illustrate the resolution of the GC-MS runs used in this study. Previous research demonstrated that average mass spectra (without the retention time dimension) contained sufficient information to enable the differentiation of truffle species (Gioacchini *et al.*, 2005) and of white truffle specimens (*T. magnatum*) collected at different places (Gioacchini *et al.*, 2008). A similar approach (i.e. proton-transfer-reaction mass spectrometry) was found to be useful with plants (Steeghs *et al.*, 2004) as long as volatile markers contained mass fragments that were unique to a single volatile in the chromatogram. Our method recorded retention time in addition to *m*/*z*; despite the short chromatographic runs, it identified volatile markers that could be traced back to a single volatile or a mixture of a few compounds.

The data of Table S2 were not normalized to sample biomass, because the variability in the samples mass (300–400 mg) only had a negligible effect on the signal intensities of the chromatograms (Fig. S2), probably because, at equilibrium, volatiles present in the headspace of the SPME vials only represented a small fraction of the volatiles trapped inside the truffle gleba. Similarly, sample storage at 4°C up to 6 d had a negligible effect on data variability compared with the variability observed among all the samples analyzed in this study (Figs S3, S4).

Because, without the use of proper internal standards to account for matrix effects, SPME-GC-MS is considered to be a semiquantitative method, we estimated to which extent matrix effects would account for variability in our data. The matrix effect on our SPME-GC-MS data was estimated for C8-VOCs with a subsample from our data set (32 truffles) by comparing the SPME-GC-MS results with those obtained by OLS-GC-FID. There was a good agreement between C8 concentrations obtained by SPME-GC-MS and the quantitative method OLS-GC-FID (*R* ^2^ = 0.86; Fig. S5), indicating that the matrix effect in SPME-GC-MS accounted for < 14% of the overall data variability. Considering only the samples where C8-VOCs could be detected, the ratios between the samples containing the maximum and minimum concentrations of C8-VOCs were 53 000% (OLS-GC-FID) and 36 000% (SPME-GC-MS; Fig. S5). The matrix effect was thus negligible compared with the variability observed in C8-VOCs concentrations among our samples. This confirms the solidity of the SPME-GC-MS data despite the fact that no internal standard was used in our study.

### Diversity in the volatiles of *T. uncinatum* fruiting bodies

Based on Table S2, four major volatile marker groups could be identified in *T. uncinatum*. These corresponded to (chemical’s name (*m*/*z*-Rt marker)): 2-butanone (*m*/*z* 72; Rt118-163); 1-octen-3-ol, 3-octanone, 3-octanol, 2-octen-1-ol, and *trans*-2-octenal (collectively named C8-VOCs (*m*/*z* 57; Rt415-523)); 2-methylbutanal and 3-methylbutanal (*m*/*z* 58; Rt109-172); and DMS (*m*/*z* 62; Rt112-136). The regional variability of these major markers is illustrated in Fig. 2.

To estimate the variability of volatile profiles on different scales (between different collection times in the same truffle orchard, between truffle orchards in the same country or between truffle orchards in Europe), we calculated a similarity index for each pair of sites and collection times. A comparison of intersite with intrasite similarity indices (*I*_VOC_) for sites that had been sampled at least four times (Wallis, Switzerland, *n* = 6 samplings; Auvergne, France, *n* = 4 samplings) revealed that VOC profiles of fruiting bodies were slightly yet significantly more similar to each other at the scale of truffle orchards than at larger geographical scales (within a country or within Europe; Table 1).

**Table 1:** Intra- and intersite similarity in volatile organic compound (VOC) profiles of *Tuber uncinatum*

	Truffle field in Wallis, Switzerland		Truffle field in Auvergne, France	
		
Similarity in samples collected at different time points	Average *I*_VOC_	± SD	Average *I*_VOC_	± SD
Within the same truffle field	0.81	0.08 a	0.82	0.08 c
Between the truffle field (Wallis or Auvergne) and other truffle fields in the same country	0.70	0.08 b	0.69	0.11 d
Between the truffle field (Wallis or Auvergne) and other truffle fields in Europe	0.68	0.12 b	0.65	0.12 d

The identity index *I*_VOC_ reflects the similarity in VOC profiles of truffles collected between two different sites or two time points. A value of 0 indicates no similarity, while a value of 1 corresponds to identical VOC profiles. Pairwise identities (*I*_VOC_) were computed between all sites/seasons listed in Supporting Information Table S2. The average pairwise identities are reported here for samplings within the sites of Wallis (Switzerland, *n* = 6 samplings) and Auvergne (France, *n* = 4) and between those sites and other sites in the same country or in Europe. Average *I*_VOC_ was significantly higher within the same site than between those sites and other sites located either in the same country or in Europe (*P* < 0.05, ANOVA and Fischer LSD *post-hoc* test; different letters indicate statistical differences for the fields of Wallis or Auvergne).

### Region-specific volatile markers produced by *T. uncinatum* fruiting bodies

Based on the fingerprint data for VOCs from all samples (Table S2), markers that significantly differed among sites were identified (Kruskall–Wallis test, *P* < 0.01) and displayed as a heat map (Fig. 3). Some markers were region-specific and can therefore serve to distinguish among samples of different sites. However, these region-specific markers did not consistently occur in all the samples collected at a single site (Fig. 3). Stepwise discriminant analysis (SDA; Gioacchini *et al.*, 2008) was applied to the data (Table S2), resulting in a model that classified 94% of all samples in their correct region of origin (Table S3). No volatile markers unique to *T. uncinatum* morphotypes *uncinatum* or *aestivum* were detected, supporting the view that these morphotypes belong to the same species (Paolocci *et al.*, 2004).

### Most of the variability in volatile profiles of five truffle species is explained by C8-VOCs

Visualization of the fingerprints of *T. uncinatum* volatiles (Table S2) using principal factor analysis revealed that variability in VOC markers was driven by quantitative differences in C8-VOCs (Fig. 4a). Specimens producing either high or low C8-VOC concentrations were detected at eight of the 14 sites (including natural truffle grounds; Fig. 4b).

1-Octen-3-ol has already been reported from numerous truffle species (Splivallo *et al.*, 2011), but its variability has not been quantified. We determined the concentration of 1-octen-3-ol from five truffle species of commercial interest by using the same method (SPME-GC-MS) already applied to *T. uncinatum*. The truffles used here had been purchased from wholesalers in France, Italy, Germany, and Hungary. Similarly to *T. uncinatum*, a high variability in 1-octen-3-ol was observed for *T. borchii*, *T. brumale*, *T. macrosporum*, and *T. indicum*, but not for *T. magnatum* (where 1-octen-3-ol could not be detected), indicating that 1-octen-3-ol is a major contributor to VOC variability in numerous truffle species (Fig. S6).

Additionally to the SPME-GC-MS data, 1-octen-3-ol and other C8-VOCs were quantified from 34 truffles originating from Switzerland, Italy, and France. 1-Octen-3-ol was by far the dominant volatile and ranged in the gleba samples (400 mg) from 0 to 23 000 ng (Fig. S5). Interestingly, the reported threshold for olfaction of 1-octen-3-ol is 10 ng g^−1^ (Combet *et al.*, 2006). Only two other C8-VOCs could be detected by OLS-GC-FID (3-octanone and 3-octanol), but they represented only a minor fraction of the quantity of 1-octen-3-ol (Fig S5). These results indicate that 1-octen-3-ol is by far the dominant C8 volatile in *T. uncinatum*.

### High/low C8-VOC producers of *T. uncinatum* cluster around specific host trees

**Fig. 5:**
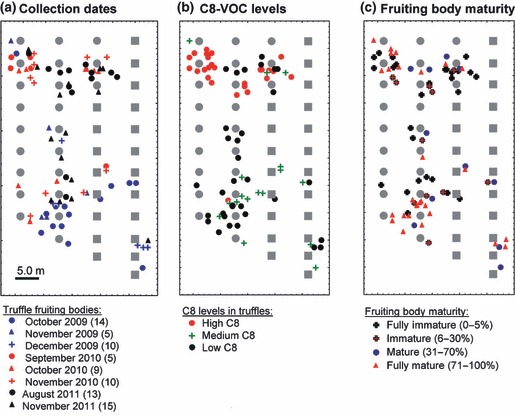
Collection date, eight-carbon volatile (C8-VOC) concentrations, and maturity of *Tuber uncinatum* fruiting bodies collected over 2 yr in a single truffle ground. Gray symbols indicate host trees (circles, hazels; squares, pines). (a) Date on which truffle fruiting bodies were collected in a truffle ground in Wallis, Switzerland. The number of fruiting bodies collected on each date is shown in brackets. In 2009, truffles were mostly collected from the lower part of the field while the opposite was true in 2010. (b) Distribution of individual fruiting bodies that produced different concentrations of C8-VOCs; fruiting bodies were grouped into three categories based on marker (*m*/*z* 57; Rt 415-523; high, > 0.40; medium, > 0.04; low, ≥ 0.00). (c) Distribution of individual fruiting bodies based on their maturity; maturity was estimated based on the percentage of ascii containing melanized spores (indicated in brackets).

To gain more insight into the variability of C8-VOCs within a single site, we sampled the same truffle orchard in Wallis (Switzerland) repeatedly over 3 yr and recorded the exact position of each sample in the field (Fig. 5a). Volatile fingerprinting revealed that high C8-VOC producers were predominantly located in the upper part of the field while medium and low producers were predominantly found in the central and lower part of the field, and clustered around specific trees (Fig. 5b). Two zones (lower and upper right parts of the field) also contained high and low C8-VOC producers located within a few cm of each other (Fig. 5b).

### Differences in fruiting body maturity do not account for differences in C8-VOC concentrations either in *T. uncinatum* or in *T. borchii*

The concentrations of most VOC markers varied greatly among fruiting bodies (Fig. 3, red arrows) and seasons (Fig. S7, blue arrows). This variability could not be explained by differences in fruiting body maturity, which was estimated from the percentage of ascii containing melanized spores (in Fig. S7; maturity represented by the upper gray bars does not correlate with the amounts of any single volatile marker).

Similarly variability in C8-VOC concentration in the *T. uncinatum* samples collected from the field in Wallis, Switzerland, was not explained by maturity (Fig. 5b,c): although numerous truffles in the upper left corner of the field produced high C8-VOC concentrations and most truffles in the lower part of the field produced medium to low C8-VOC concentrations, maturity in both parts of the field ranged from immature to fully mature. A linear regression model confirmed that fruiting body maturity and C8-VOC concentration (reflected by marker (*m*/*z* 57.0; RT415-523)) were not correlated (*P* = 0.23, *R* ^2^ = 0.028). 1-Octen-3-ol was believed to occur only in fully mature (71–100% maturity) fruiting bodies of *T. borchii* (Zeppa *et al.*, 2004). In the same truffle species we could, however, detect 1-octen-3-ol in specimens that were 31–70% mature. Furthermore, the same volatile was also undetectable from some fully mature samples (71–100% maturity, Table S4), which contradicts the claim that 1-octen-3-ol is a marker for fully ripe truffles (Zeppa *et al.*, 2004).

### C8-VOC concentrations are related to truffle genotype in *T. uncinatum*

**Fig. 6:**
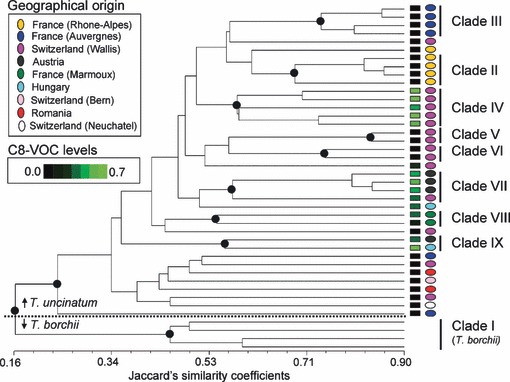
Relationship between eight-carbon volatile (C8-VOC) concentrations and genetic background. Dendogram based on the amplified fragment length polymorphism (AFLP) data for four samples of *Tuber borchii* (two mycelium and two fruiting bodies) and 42 samples of *Tuber uncinatum*. Black circles indicate nodes > 80% (2000 bootstraps). C8-VOC concentrations and geographical region for samples forming a clade are color-coded. C8-VOC concentrations were determined for *T. uncinatum* only and not for *T. borchii* samples.

Because fruiting body maturity and geographical origin failed to explain differences in C8-VOC concentrations, we used AFLP to further investigate whether differences in genotype accounted for the variability in C8-VOCs. AFLP is a genomic fingerprinting method that is based on dominant markers and that has been widely used with plants, bacteria, and fungi (Vos *et al.*, 1995; Bensch & Akesson, 2005), including numerous ectomycorrhizal species (Douhan *et al.*, 2011). We successfully generated DNA that was completely digestible with both restriction endonucleases used in the AFLP analysis for 42 samples originating from nine geographical regions. AFLP fingerprints revealed that *T. uncinatum* truffles collected at a single time point and at the same place often clustered as one clade/genotype, and that genotype was related to C8-VOC concentrations (Fig. 6). This suggests that the capacity to produce C8-VOCs is genetically determined. For the 15 samples collected from the truffle field in Switzerland, the AFLP data highlight that different genotypes were located in distinct parts of the field. Also, these data support the hypothesis that genotypes differ in their capacity to produce C8-VOCs (Fig. S8).

### 1-Octen-3-ol production capacity of *T. borchii* is strain-specific

The AFLP data demonstrate that C8-VOC concentrations are related to genetic background. Because the samples used to generate the AFLP data were collected at different places and times, the influence of other biotic (e.g. host plant, microbes) or abiotic factors (e.g. soil temperature, soil fertility) on C8-VOC production cannot be completely ruled out. It is also possible that soil conditions were heterogeneous in the truffle ground of Wallis (Switzerland) and specifically in the zones where high and low C8-VOC producers were located. To determine whether C8-VOC production differed among truffle genotypes in the absence of environmental variability, we conducted a controlled laboratory experiment. We selected two strains of *T. borchii* that were isolated from the same Italian field (Bonuso *et al.*, 2009) and one strain of *T. melanosporum* (strain TN1 provided by Dr G. Chevalier, INRA Clermont-Ferrand, France) and quantified their C8-VOC production under controlled laboratory conditions. Of the C8-VOCs, only 1-octen-3-ol could be detected. The two strains of *T. borchii* differed drastically in their production of 1-octen-3-ol, the major C8-VOC (Fig. S8). These results support the inference that the capacity to produce C8-VOC within the same truffle species is strain-specific.

## Discussion

The present study demonstrates that C8-VOCs are major volatiles of black and white truffle species and contribute to intraspecific aroma variability in the black species *T. uncinatum*. The study also shows that in *T. borchii* and *T. uncinatum*, the dominant C8-VOC, 1-octen-3-ol, does not strictly occur in fully mature truffles as argued earlier (Zeppa *et al.*, 2004). This C8-VOC was undetectable in some fully mature fruiting bodies and was detectable in some immature fruiting bodies, demonstrating that its biosynthesis is not maturity-dependent (Fig. 5).

### An ecological role for C8-VOCs in truffles?

The physiological and ecological roles of the major C8-VOC, 1-octen-3-ol, are speculative for truffles. In other fungi, this volatile acts as a sexual hormone (Chitarra *et al.*, 2004; Tsitsigiannis *et al.*, 2005) and also attracts numerous insect species (Combet *et al.*, 2006). In truffles, 1-octen-3-ol might attract the fly *Suillia pallida* or the beetle *Leiodes cinnamomea* to fruiting bodies (Hochberg *et al.*, 2003; Maser *et al.*, 2008). Whether these insects act as pests or whether they are beneficial for the fungus (i.e. for spores’ dispersal) is unknown. 1-Octen-3-ol might also be involved in communication with plant roots and influence the fitness of host/nonhost plants (Splivallo *et al.*, 2007b, 2011). Estimating the overall cost–benefit for truffles for producing varying amounts of 1-octen3-ol and understanding its ecological role will be a major challenge, considering that truffle fruiting bodies cannot be obtained under laboratory conditions.

### Do distinct C8-VOC concentrations in truffles result from a mutation?

Rather than indicating that variation in C8-VOC synthesis is a result of differences in maturity, our data indicate that variation in C8-VOC synthesis likely reflects genotypic differences among strains. A comparable genotypic determinant of volatile synthesis has been reported for strawberries; because of an insertional mutation affecting the expression of a terpene synthase gene, wild strawberries (*Fragaria* spp.) produce different isoprenoids from those produced by cultivated species (Aharoni *et al.*, 2004). Whether a similar mutation or another mechanism affects C8-VOC biosynthesis in truffles is still unclear, and will require, as in other fungi (Brodhun *et al.*, 2009; Garscha & Oliw, 2009), understanding the C8-VOC biosynthesis pathway. However, because a single gene is responsible for C8-VOC biosynthesis in the model fungus *Aspergillus nidulans* (Brodhun *et al.*, 2009), it is possible that polymorphisms in the homologous gene in *T. borchii* and *T. uncinatum* are responsible for the observed intraspecific aroma variability. The genome of the black truffle *T. melanosporum* contains two putative candidate genes (gene model GSTUMT00000322001 annotated as TmelPpo1 and gene model GSTUMT00006891001 annotated as TmelPpo2) that are potentially involved in the synthesis of 1-octen-3-ol (Martin *et al.*, 2010).

### Are C8-VOCs linked to truffle sexuality?

Repeated sampling of the same productive patches from a truffle orchard in Switzerland revealed that the aroma of truffles collected from the same patch at different times was very similar in terms of C8-VOCs. These observations strongly support recent insights into the sexuality of the black Périgord truffle. In *T. melanosporum*, spores are made up of parents of opposite mating types (Martin *et al.*, 2010; Rubini *et al*., 2011a, b). The spores, contained in ascii, are surrounded by aggregated hyphae (the gleba) originating from a single parent (the mother) (Rubini *et al.*, 2011a). The mother strain also colonizes the roots of the host tree and forms a below-ground genet, which explains why the gleba tissue of fruiting bodies surrounding the same host tree is genetically identical (Rubini *et al.*, 2011a).

Similarly to the findings of Rubini *et al.* (2011a), our AFLP data revealed high similarities among truffles collected from the same sites and, for the site in Wallis (Switzerland), for truffles collected from around the same host trees. In contrast to what has been observed with *T. melanopsorum*, the fruiting bodies of *T. uncinatum* surrounding a single host tree (Wallis field) did not display clonal identity but formed significant AFLP clades with 68–83% similarity (Jaccard’s coefficient) (Fig. 6). These apparent differences with *T. melanosporum* can be explained in three ways. First, fruiting bodies sharing the same ‘mother’ might have different ‘fathers’, as already demonstrated for *T. melanopsorum* (Rubini *et al.*, 2011a). Because standard DNA extraction predominantly yields the DNA of the ‘mother’ only for fully mature truffles (Paolocci *et al.*, 2006; Rubini *et al.*, 2011a), and because our samples varied in maturity (Fig. 5), the extracted DNA of our samples was a mixture of DNA from both parents. This would explain why fruiting bodies with the same ‘mother’ but with different ‘fathers’ would produce somewhat different AFLP fingerprints. Second, as in other ectomycorrhizal species (Douhan *et al.*, 2011), genets formed by *T. uncinatum* might be unstable in space and time, resulting in highly similar yet nonclonal AFLP fingerprints. Third, some signals in the AFLP fingerprints could be the result of microbial contaminates in the fruiting bodies (Barbieri *et al.*, 2005, 2007; Buzzini *et al.*, 2005; Pacioni *et al.*, 2007), and such contaminants may have differed among samples.

By analogy to other fungi where 1-octen-3-ol acts as a sexual hormone (Chitarra *et al.*, 2004; Tsitsigiannis *et al.*, 2005), in truffles, high and low amounts of 1-octen-3-ol might be linked to mating types. In this speculative case, 1-octen-3-ol could be involved in the recognition of opposite mating types before sexual reproduction. This hypothesis should be tested in *T. melanopsorum* considering that mating types have already been identified for that truffle species (Martin *et al.*, 2010; Rubini *et al.*, 2011b).

### Are C8-VOCs produced by truffles or associated microbes?

The production of C8-VOCs by microbes inhabiting the rhizosphere and colonizing truffles or an indirect induction of C8-VOCs in truffle fruiting bodies caused by the presence of specific microbes cannot be fully excluded. The slight changes in volatile profiles of *T. uncinatum* observed upon storage at 4°C (Figs S3a, S4) can most likely be attributed to changes in metabolism of the fungus itself and also to microbial growth typically observed with stored food. By contrast, storage did not influence C8-VOC concentrations of *T. uncinatum* (Fig. S3b).

Determining if soil microbial community or the microbial community trapped inside truffle fruiting bodies (Barbieri *et al.*, 2005, 2007; Mello *et al.*, 2010) has an influence C8-VOC emission will require an understanding of the population structure of microbes associated with truffle fruiting bodies as well as an investigation into what volatiles these microbes are able to produce. The data generated with *T. borchii* mycelium nevertheless suggest that the capacity to produce 1-octen-3-ol varies among truffle strains. Indeed two strains of *T. borchii* originating from the same Italian truffle ground (Bonuso *et al.*, 2009) produced different amounts of 1-octen-3-ol (Fig. S9), even though they were colonized by the same bacterial endophyte (Barbieri *et al.*, 2000, 2002).

### Influence of biotic and abiotic factors on truffle aroma

Our results also demonstrate that the influence of the soil and climate on truffle aroma cannot be studied without considering the influence of genotype. Gioacchini *et al.* (2008) suggested that some isoprenoids such as cedrol and himachalene should be considered as markers for white truffles (*T. magnatum*) from the Piedmont (cedrol) or Umbria (himachalene) regions, but these authors failed to consider the genotypic variability of their samples, nor did they show that these markers consistently occurred in truffles collected from one year to the next.

Estimating the contribution to truffle aroma variability of the genotype, of environmental factors (i.e. soil physicochemical properties, microbes, host tree), and of physiological factors (i.e. maturation, aging) will require a holistic approach, monitoring all variables for a large number of samples. Most of all this should be performed using truffles of the same genotypes (same mother and father), which might require numerous years considering the low and variable yield of truffles typical of truffle orchards.

### A first step towards quality control and consumer preference

Differences in C8-VOC content might affect olfactory notes and thus consumer preferences, as illustrated with the pine-mushroom *Tricholoma matsutake* (Cho *et al.*, 2008). In the case of *T. uncinatum* (morphotype *aestivum*), aroma compounds sensed by humans include three sulfur-containing compounds (including DMS, which was identified as a major volatile of *T. uncinatum*; Fig. 2), as well as two alcohols and one ketone (Culleré*et al.*, 2010). In the study by Culleré*et al.* (2010), 1-octen-3-ol did not affect the aroma of the samples probably because its concentration in those Spanish truffles was low (Culleré*et al.*, 2010). In a number of our samples, however, 1-octen-3-ol concentrations were above its threshold of olfaction. It might consequently influence the overall aroma perception by humans. Understanding how 1-octen-3-ol affects product quality will require the establishing of sensory panels and the testing of a large number of truffle samples, considering the large aroma variability observed among fruiting bodies of the same species.

In summary, we have shown that variation in C8-VOC biosynthesis in truffles is linked to genotypic variation. Given that most of the fruiting body biomass is represented by the tissue surrounding the spores (the gleba) rather than the spores themselves (Paolocci *et al.*, 2006; Rubini *et al.*, 2011a), and assuming that the amount of VOCs produced per unit of gleba and spore biomass are equal, gleba VOCs would dominate the overall VOC profiles of fruiting bodies. Distinguishing the contribution of each parent or the contribution of biotic/abiotic factors to truffle aroma will require fruiting bodies to be obtained from known parents of opposite mating type under different conditions (i.e. soil, climate), a major challenge considering the difficulty in producing fruiting bodies under controlled conditions.

## Supporting Information

**Fig. S1** Chromatograms of *Tuber uncinatum* from different countries.

**Fig. S2** Effect of sample mass on volatile fingerprinting by SPME-GC/MS.

**Fig. S3** Storage of truffles at 4°C upto 6 d has a minor influence on volatile profiles.

**Fig. S4** Chromatograms of truffles producing low and high C8 volatiles stored at 4°C up to 6 d.

**Fig. S5** Comparing SPME-GC/MS and OLS-GC/FID data sets to estimate the matrix effect.

**Fig. S6** Variability in 1-octen-3-ol in truffles.

**Fig. S7** Season variability of volatile markers within single sites.

**Fig. S8** Distribution in the field of Wallis (Switzerland) of the different genotypes (based on AFLP data).

**Fig. S9** 1-Octen-3-ol intra and interspecific variability in mycelia of *Tuber borchii* and *Tuber melanosporum*.

**Table S1** Detailed information for *Tuber uncinatum* fruiting body samples

**Table S2** Raw data for chromatogram fingerprints for volatiles of *Tuber uncinatum*

**Table S3** Model based on discriminant analysis classifies 94% of all *Tuber uncinatum* samples in the correct region of origin

**Table S4** 1-Octen-3-ol biosynthesis in *Tuber borchii* is not maturity dependent
