# Supplementary material for: Intraspecific genotypic variability determines concentrations of key truffle volatiles
Source: New Phytol. 2012 May;194(3):823–35. doi: 10.1111/j.1469-8137.2012.04077.x (PMC3470932; doi:10.1111/j.1469-8137.2012.04077.x)
Supplement: Supplementary file 1 [file nph0194-0823-SD1.pdf]

## Supporting Information Figs S1-S9 & Tables S1, S3, S4

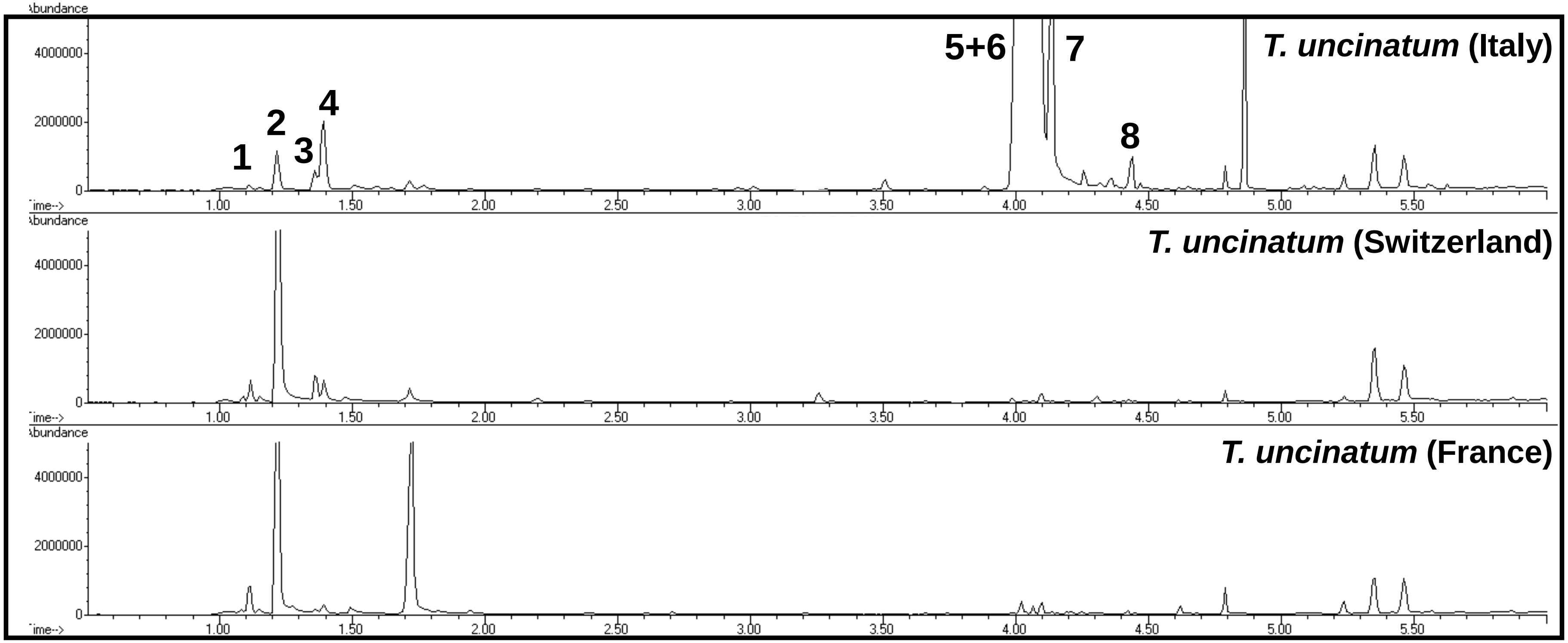


**Fig. S1. Chromatograms of *Tuber uncinatum* from different countries.**

Chromatograms (TIC) were obtained by SPME-GC/MS. Major volatiles (detected in >65% of all samples analyzed in this study) of *T. uncinatum* are shown (chemical´s name and corresponding marker): **(1)** Dimethyl sulfide [m/z62; Rt112-136] ; **(2)** 2-butanone [m/z72; Rt118-163]; **(3)** 3-methyl-1-butanal and **(4)** 2-methyl-1-butanal – (3&4) together [m/z58; Rt109-172]; **(5 + 6)** 1-octen-3-ol and 3-octanone; **(7)** 3-octanol; **(8)** trans-2-octenal - (5+6+7+8) together [m/z57; Rt415-523]

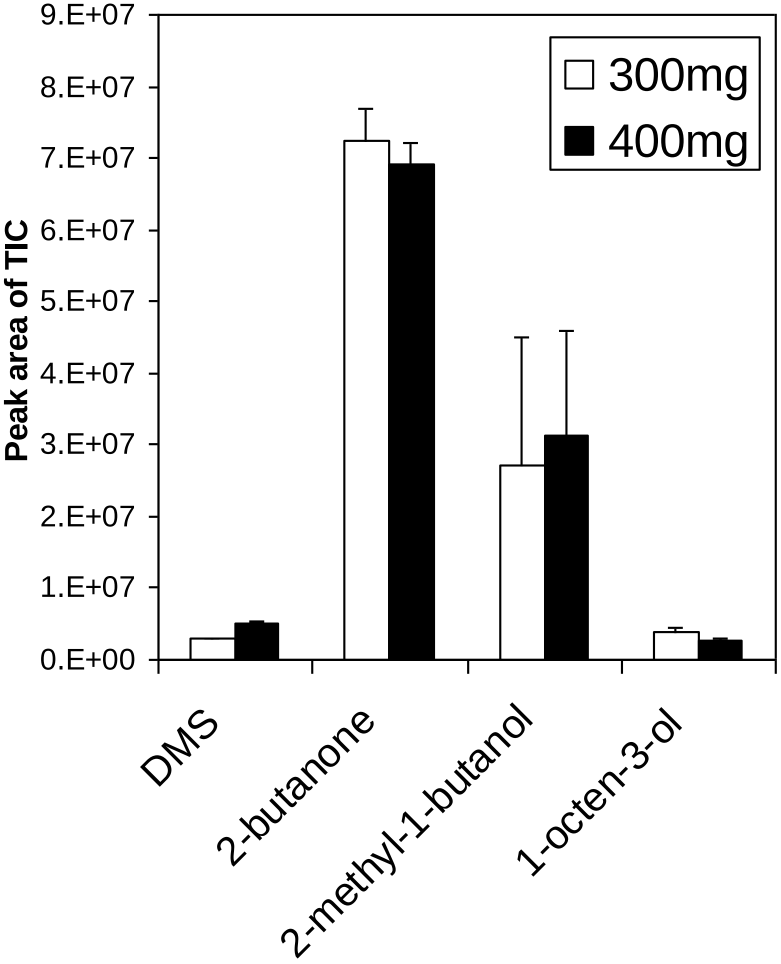


**Fig. S2 Effect of sample mass on volatile fingerprinting by SPME-GC/MS.**

Under equilibrium extraction with SPME, samples of 300 mg or 400 mg only presented a negligible variability in terms of peak intensity (shown here for 4 major volatiles, *n* = 3 replicates). This variability was comparable to the variability observed in between different gleba samples of the same truffle, but negligible compared to the variability observed among all the *Tuber uncinatum* samples analyzed in this study (i.e. 1-octen-3-ol TIC peak area ranged from zero to $1 \times 10^9$).

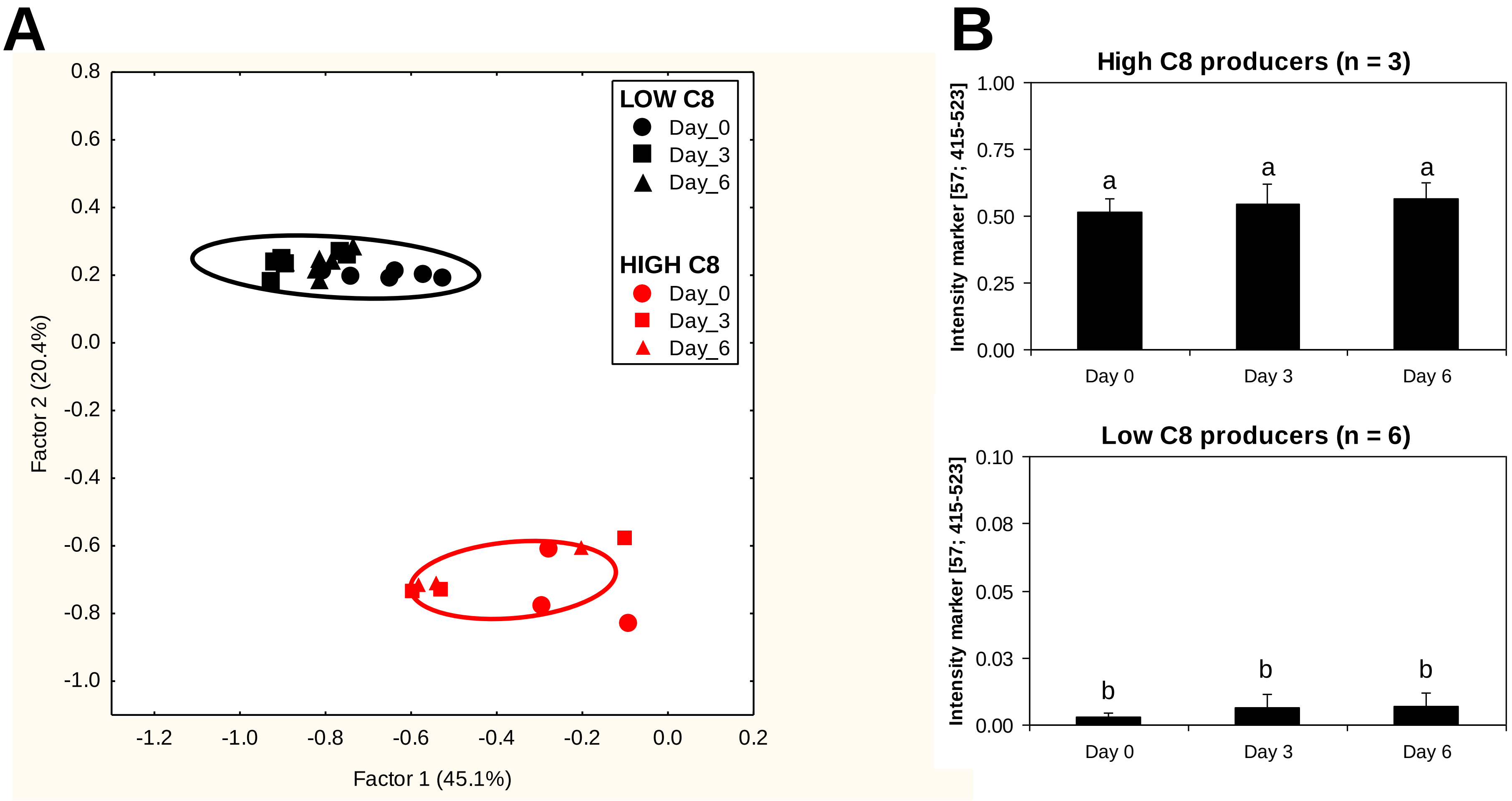


**Fig. S3 Storage of truffles at 4°C up to 6 d has a minor influence on volatile profiles.**

**(A)** To test the effect of storage on truffle aroma, nine truffles collected from a field in Wallis (Switzerland) were subdivided in three pieces right after collection. The first piece was immediately frozen (Day_0), the second and third pieces were stored at 4°C and frozen after 3 d (Day_3) and 6 d (Day_6). Volatile profiles were generated for all samples (0.40 ± 0.02 g of gleba) by SPME-GC/MS. The scatterplot **(A)** illustrates factor loadings (principal factor analysis) with 95% confidence ellipses. Samples were grouped in two categories based on marker [m/z57; Rt 415-523] reflecting the occurrence of C8-VOCs (1.00 ≥ high > 0.40; 0.04 ≥ low ≥ 0.00). Aging up to 6 d at 4°C only had a minor effect on the volatile profiles of the samples. **(B)** C8 volatiles (normalized intensities of marker [m/z57; Rt 415-523]) did not change significantly upon storage, either in truffles producing high C8 or low C8 ($P > 0.05$; Kruskal-Wallis test).

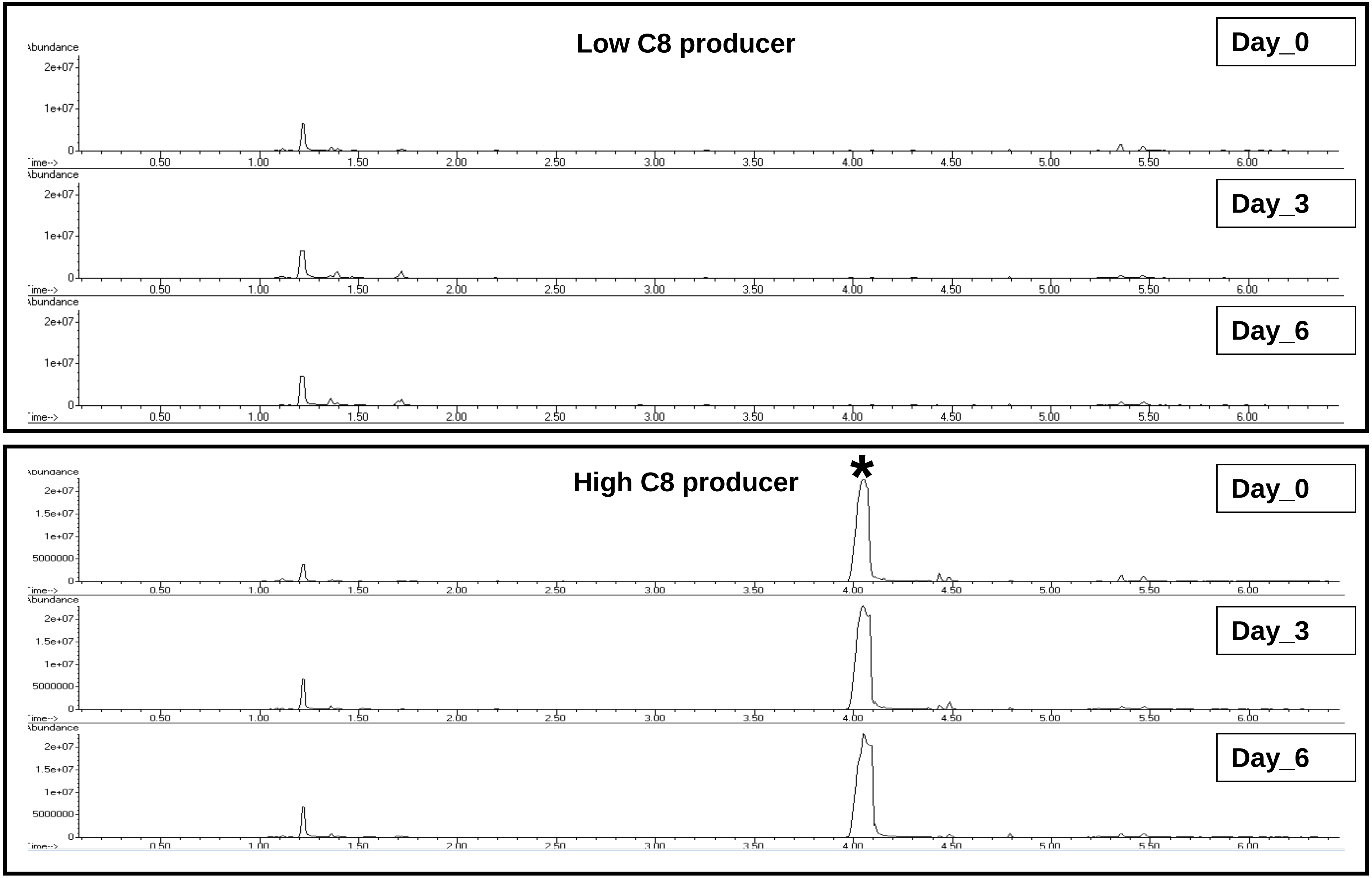


**Fig. S4 Chromatograms of truffles producing low and high C8 volatiles stored at 4˚C up to 6 d.**
Chromatograms (TIC) were obtained by SPME-GC/MS. The asterix (*) refers to C8 volatiles (marker [m/z57; Rt 415-523]). Chromatograms are shown using the same y-axis scale to highlight the dominance of the C8 volatiles in the truffle producing high C8 levels.  Aging at 4˚C up to 6 d has no major effect on the the volatile profile of truffles.

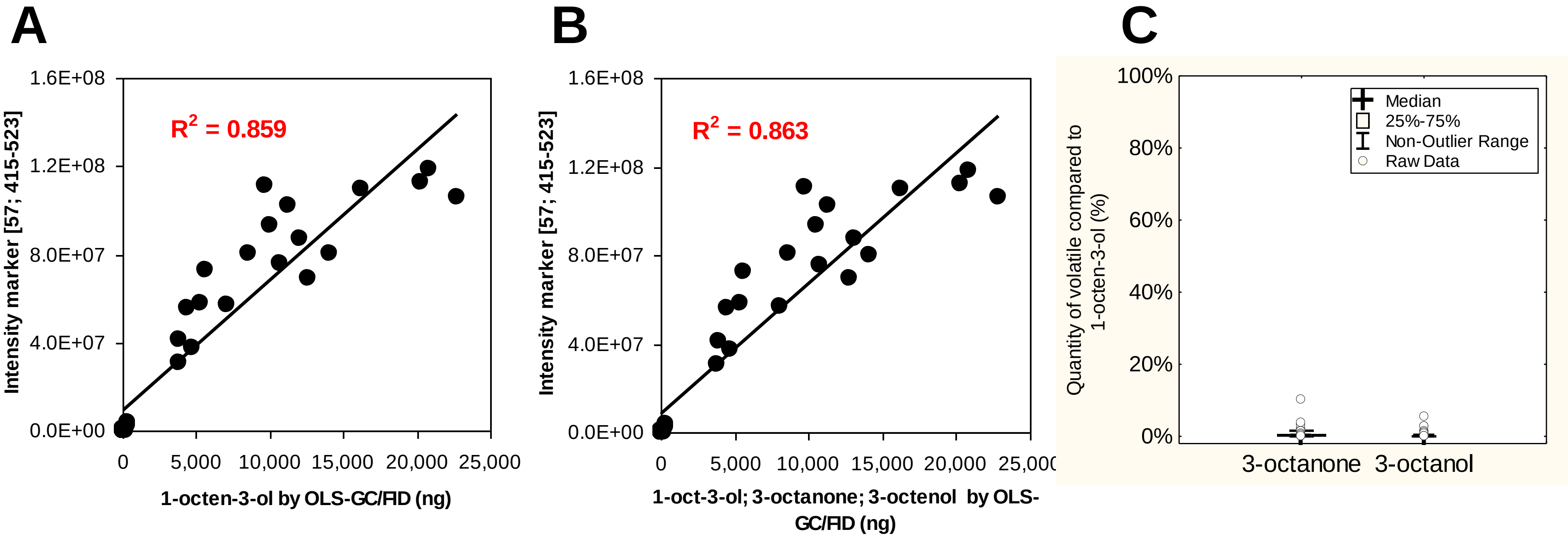


**Fig. S5. Comparing SPME-GC/MS and OLS-GC/FID data sets to estimate the matrix effect**

A total of 32 *Tuber uncinatum* samples (400 mg gleba) from Switzerland, Italy and France were analyzed by SPME-GC/MS (no internal standard) and OLS-GC/FID (with internal standard) for C8-VOC. 1-Octen-3-ol, 3-octanone and 3-octanol could be detected with the OLS-GC/FID. **(A)** Comparing both methods highlights a good agreement ($R^2 = 0.859$) between the intensities of the C8-VOC marker [m/z 57; Rt415-523] (SPME data) and the quantification of 1-octen-3-ol (OLS data). **(B)** Comparing the SPME data to the sum of all C8-VOCs detected by with the OLS further improved the correlation between both methods ($R^2 = 0.863$). This indicates that the matrix effect is negligible compared to the variability observed in the *T. uncinatum* samples. **(C)** Of the three C8-VOCs detected by OLS-GC/FID, 1-octen-3-ol was by far the dominant one; 3-octanone and 3-octanol only represented a minor fraction of the quantity of 1-octen-3-ol (max. 10%, median <1%).

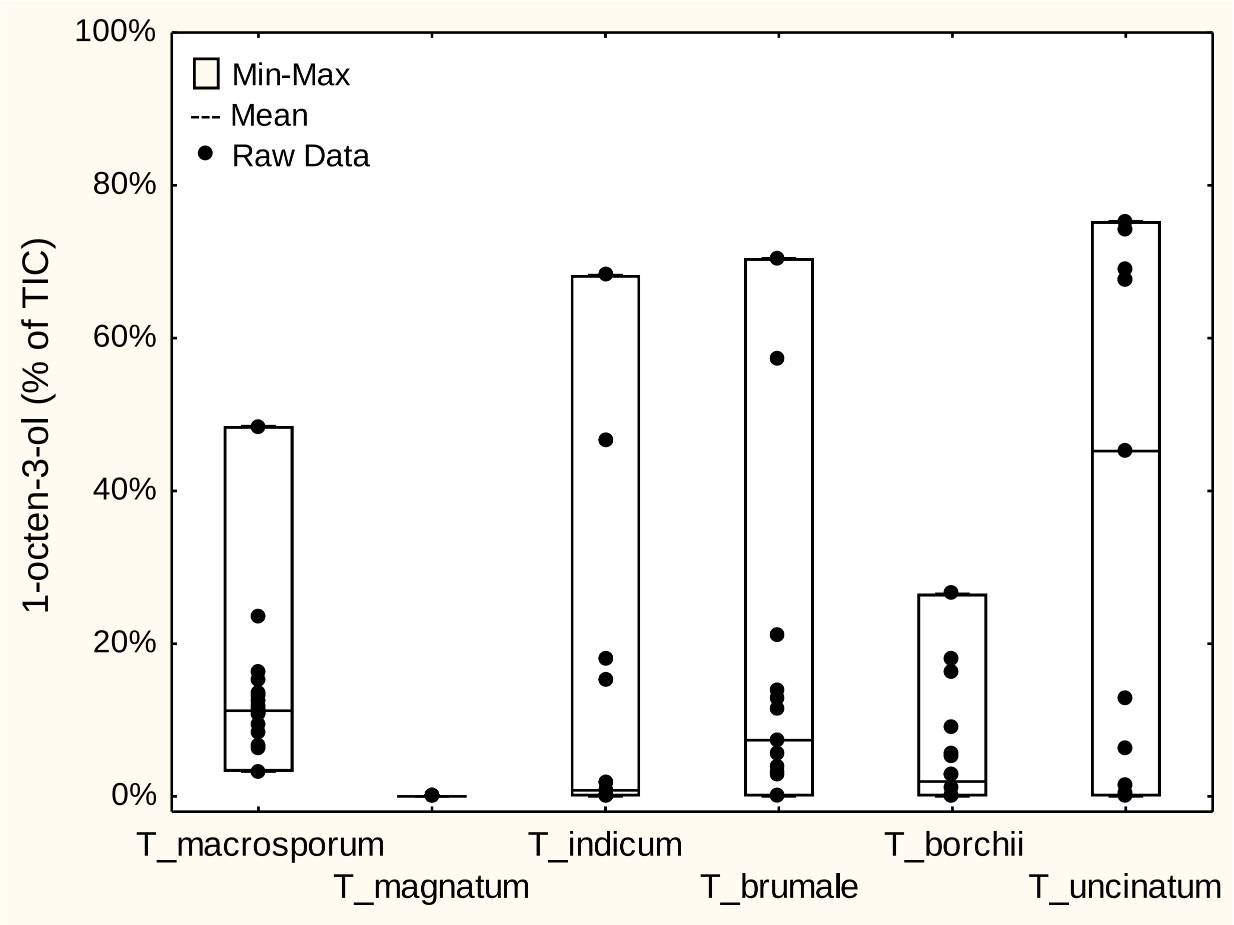


**Fig S6 Variability in 1-octen-3-ol in truffles.**

Levels of 1-octen-3-ol in six truffle species by SPME-GC/MS (11 to 18 fruiting bodies were analyzed per species). Values are expressed as peak area percent of the total ion chromatogram. 1-Octen-3-ol could be detected in all species except the white truffle *Tuber magnatum*. Note the important variability in 1-octen-3-ol within a single species.

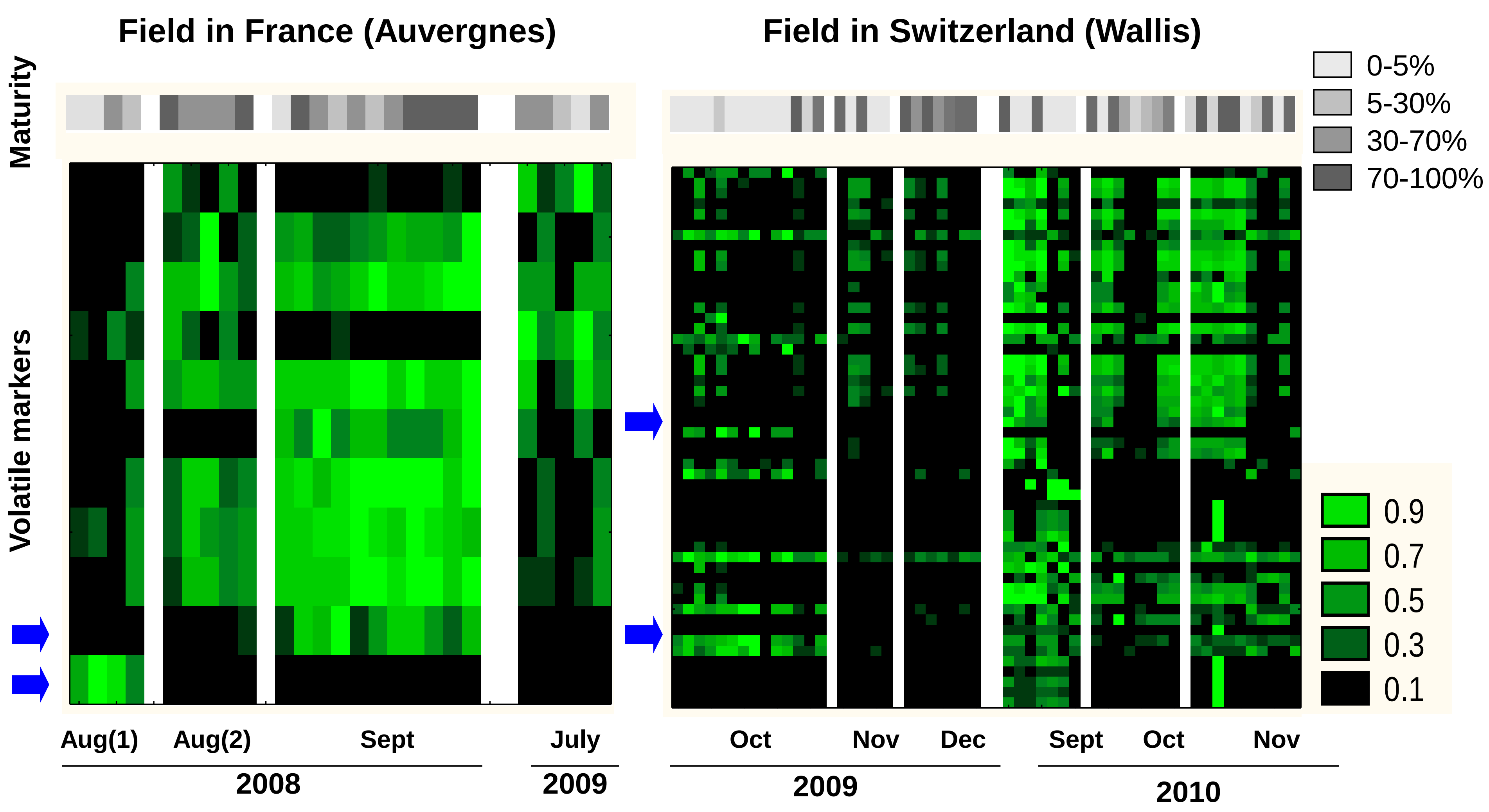


**Fig S7 Season variability of volatile markers within single sites.**

The heatmaps above were generated from Table S1 by selecting volatile markers that presented significant seasonal differences for the sites in France (Auvergnes, *n* = 4 collection times) and Switzerland (Wallis, *n* = 6 collection times) ($P < 0.01$, Kruskal Wallis test). Markers indicated with an arrow (from the top of the figure) Field in France [m/z91; Rt336-364] =1,3-dimethylbenzene + ethylbenzene; [m/z91; Rt214-254]= toluene; Field in Switzerland: [m/z98; Rt486-510]= 2-octen-1-ol + t-2-octenal, [m/z50;Rt119-167]= 2-butanone+2&3-methylbutanal. Maturity for each fruiting bodies is colour coded in grey scale and illustrates that seasonal variability in VOC profile cannot be accounted for by differences in maturity among the samples.

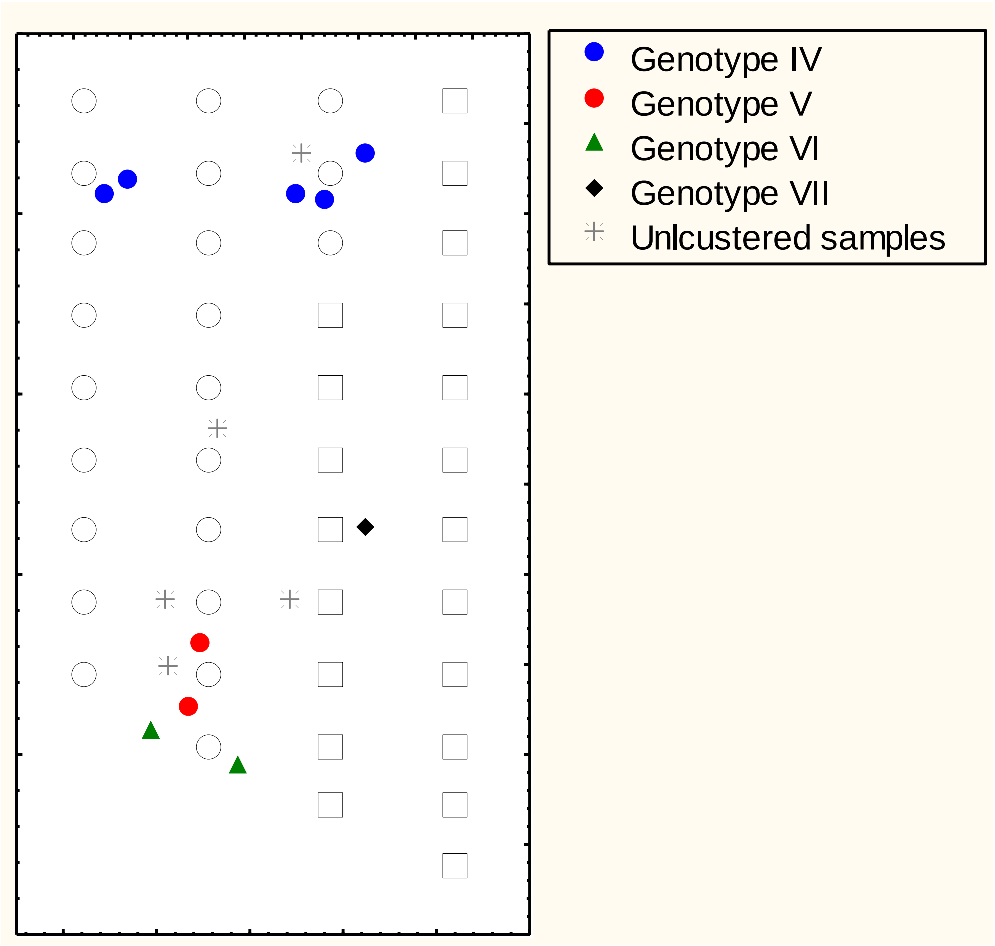


**<u>Fig S8 Distribution in the field of Wallis (Switzerland) of the different genotypes (based on AFLP data).</u>**

Distribution of genotypes in the Wallis field. For the genotype/clade numbers refer Fig 6.

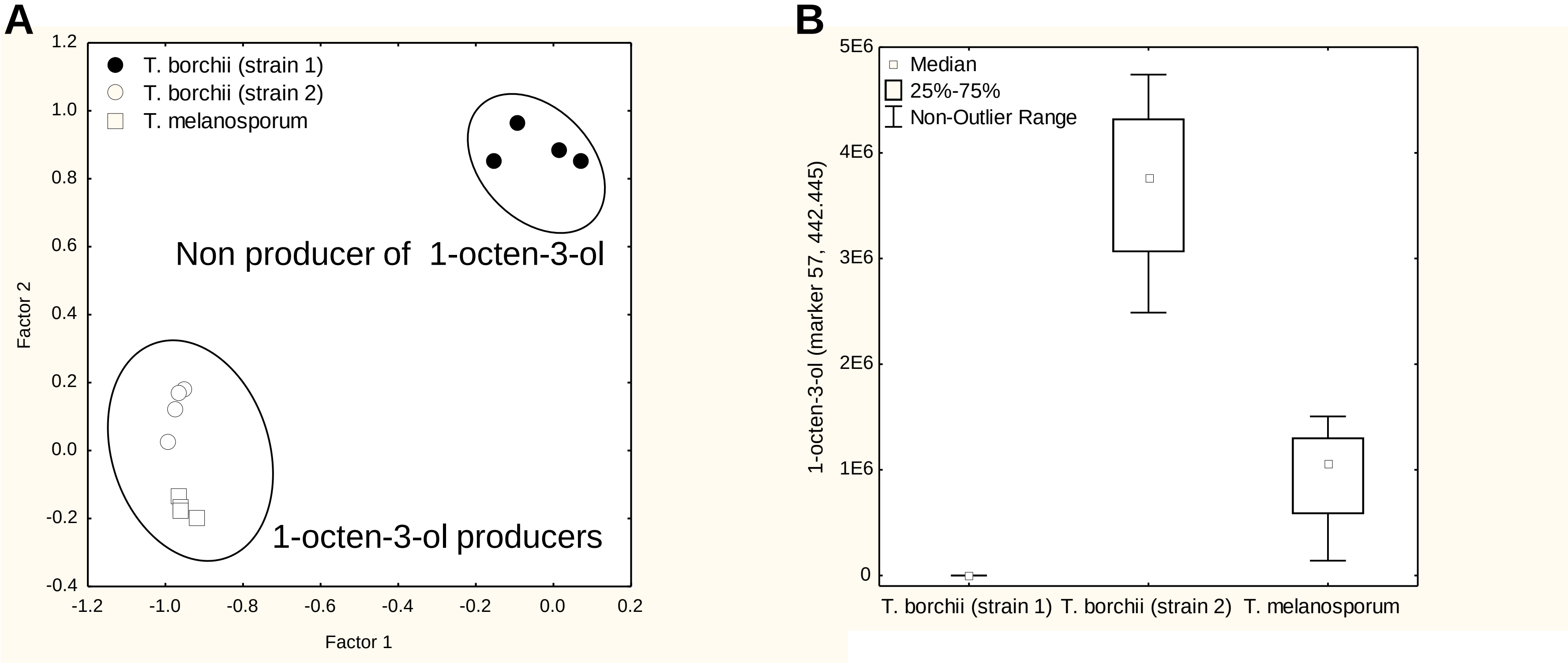


**Fig S9 1-Octen-3-ol intra and interspecific variability in mycelia of *Tuber borchii* and *T. melanosporum*.**
**(A)** Scatterplot of factor loadings (principal factor analysis) of the volatile profiles of mycelia of *T. borchii* (two strains) and *T. melanopsorum* (one strain) indicates that C8 producers can be distinguished from non producers. **(B)** Boxplot of the the major C8 volatile 1-octen-3-ol (4 replicates per strain) highlights differences in C8 levels between the two isolates of *T. borchii*, confirming the strain specificity of C8 biosynthesis.

| Samples code | Country of Origin | Closest City of Origin | Collection Year | Collection Month | Nb of fruitingbodies | Natural or artificial truffle ground | Host tree (ND= not determined) |
|---|---|---|---|---|---|---|---|
| UNC_TP_11/2009 | Austria | Berndorf | 2009 | November | 11 | Natural | Q.cerrisand, Q. robur, P.sylvestris, Carpinus betulus, Picea spp |
| UNC_GC_2_08/2008 | France | Auvergnes | 2008 | August | 5 | Artificial | Quercus spp , Corylus spp, Ostrya carpinifolia |
| UNC_GC_09/2008 | | | 2008 | September | 11 | Artificial | Quercus spp , Corylus spp, Ostrya carpinifolia |
| UNC_GC_08/2008 | | | 2008 | August | 4 | Artificial | Quercus spp , Corylus spp, Ostrya carpinifolia |
| UNC_GC_07/2009 | | | 2009 | July | 5 | Artificial | Quercus spp , Corylus spp, Ostrya carpinifolia |
| AEST_GC06/2008 | | Beauchien | 2008 | June | 8 | Artificial | Q. ilex |
| AEST_DM_07_2009 | | | 2009 | July | 5 | Artificial | Q. ilex |
| AEST_GC_08/2009 | | Mauroux | 2009 | August | 4 | ND | Quercus robur x petraea |
| UNC_GC_06/2010 | | Montélimar | 2010 | June | 8 | Artificial | Quercus ilex |
| AEST_AY_07/2008 | | Grignan - site 1 | 2008 | July | 7 | Artificial | ND |
| AEST_AY_07/2009 | | Grignan - site 2 | 2009 | July | 7 | Artificial | ND |
| UNC_GC_01/2009 | | Verdun | 2009 | January | 5 | Natural | Corylus spp |
| AEST_IB(2)_11/2009 | Hungary | Gant | 2009 | November | 9 | Natural | Quercus robur |
| UNC_TL_10/2009 | Italy | Val Curone | 2009 | October | 12 | ND | ND |
| AEST_IB(1)_11/2009 | Romania | Sepsiszentgyörgy | 2009 | November | 9 | Natural | Fagus silvatica |
| UNC_MG(1)_10_2009 | Switzerland | Bern | 2009 | October | 6 | Natural | ND |
| UNC_MG(2)_10_2009 | | Neuchatel | 2009 | October | 9 | Natural | Quercus spp and Fagus spp |
| UNC_JPS_10_2009 | | Wallis | 2009 | October | 14 | Artificial | Pinus nigra and Quercus robur |
| UNC_JPS_11_2009 | | | 2009 | November | 5 | Artificial | Pinus nigra and Quercus robur |
| UNC_JPS_12_2009 | | | 2009 | December | 10 | Artificial | Pinus nigra and Quercus robur |
| UNC_JPS_09/2010 | | | 2010 | September | 5 | Artificial | Pinus nigra and Quercus robur |
| UNC_JPS_10/2010 | | | 2010 | October | 9 | Artificial | Pinus nigra and Quercus robur |
| UNC_JPS_11/2010 | | | 2010 | November | 10 | Artificial | Pinus nigra and Quercus robur |
| UNC_JPS_08/2011 | | | 2011 | August | 13 | Artificial | Pinus nigra and Quercus robur |
| UNC_JPS_11/2011 | | | 2011 | Novemver | 15 | Artificial | Pinus nigra and Quercus robur |
| UNC/AEST_MAF_09/ | United Kingdom | ND | 2008 | September | 7 | Natural | ND |
| UNC_MAF_01/2010 | | ND | 2010 | January | 11 | Natural | ND |

**Table S1 Detailed specifications about *Tuber uncinatum* fruiting bodies samples**

ND, not determined.

# Regions can be distinguished by stepwise discriminant analysis

| | | | ***Predicted nb of samples in each regions*** | | | | | | | | | | | | | |
|---|---|---|---|---|---|---|---|---|---|---|---|---|---|---|---|---|
| | Total Nb of samples | Samples correctly Classification (%) | South FR | South West FR | South East FR (1) | South East FR (2) | North East FR | Central FR | South Switzerland | Central Switzerland | North West Switz. | UK | Italy | Austria | Hungary | Romania |
| South France | 13 | **85%** | 11 | 0 | 0 | 0 | 0 | 0 | 2 | 0 | 0 | 0 | 0 | 0 | 0 | 0 |
| South West France | 4 | **75%** | 0 | 3 | 0 | 0 | 0 | 0 | 1 | 0 | 0 | 0 | 0 | 0 | 0 | 0 |
| South East FR (1) | 14 | **100%** | 0 | 0 | 14 | 0 | 0 | 0 | 0 | 0 | 0 | 0 | 0 | 0 | 0 | 0 |
| South East FR (2) | 8 | **100%** | 0 | 0 | 0 | 8 | 0 | 0 | 0 | 0 | 0 | 0 | 0 | 0 | 0 | 0 |
| North East France | 5 | **100%** | 0 | 0 | 0 | 0 | 5 | 0 | 0 | 0 | 0 | 0 | 0 | 0 | 0 | 0 |
| Central France | 25 | **100%** | 0 | 0 | 0 | 0 | 0 | 25 | 0 | 0 | 0 | 0 | 0 | 0 | 0 | 0 |
| South Switzerland | 55 | **98%** | 1 | 0 | 0 | 0 | 0 | 0 | 54 | 0 | 0 | 0 | 0 | 0 | 0 | 0 |
| Central Switzerland | 6 | **100%** | 0 | 0 | 0 | 0 | 0 | 0 | 0 | 6 | 0 | 0 | 0 | 0 | 0 | 0 |
| North West Switz. | 9 | **100%** | 0 | 0 | 0 | 0 | 0 | 0 | 0 | 0 | 9 | 0 | 0 | 0 | 0 | 0 |
| UK | 18 | **78%** | 0 | 0 | 0 | 0 | 0 | 0 | 2 | 2 | 0 | 14 | 0 | 0 | 0 | 0 |
| Italy | 12 | **83%** | 0 | 0 | 0 | 0 | 0 | 0 | 0 | 2 | 0 | 0 | 10 | 0 | 0 | 0 |
| Austria | 11 | **100%** | 0 | 0 | 0 | 0 | 0 | 0 | 0 | 0 | 0 | 0 | 0 | 11 | 0 | 0 |
| Hungary | 10 | **100%** | 0 | 0 | 0 | 0 | 0 | 0 | 0 | 0 | 0 | 0 | 0 | 0 | 10 | 0 |
| Romania | 9 | **89%** | 0 | 0 | 0 | 0 | 0 | 0 | 0 | 0 | 1 | 0 | 0 | 0 | 0 | 8 |
| **Avg Correct Classification** | | **94%** | | | | | | | | | | | | | | |

**Table S3 Model based on discriminant analysis of volatile fingerprints classifies 94% of all samples in the correct region of origin.**

Discriminant analysis was performed on Table S2 using stepwise forward discriminant analysis ($F$ to remove = 0.05; $F$ to enter = 0.1). Calssification scores and the number of samples in each regions are listed in the table above.

| Sample number | 1-octen-3-ol | Fruiting body maturity |
|---|---|---|
| 1 | nd | II |
| 2 | √ | III |
| 3 | nd | III |
| 4 | nd | I |
| 5 | √ | II |
| 6 | nd | II |
| 7 | nd | III |
| 8 | √ | III |
| 9 | √ | II |
| 10 | nd | III |
| 11 | nd | II |
| 12 | √ | III |
| 13 | √ | II |
| 14 | √ | II |
| 15 | √ | III |
| 16 | √ | II |

**Table S4 1-Octen-3-ol biosynthesis in *Tuber borchii* is not maturity dependent.**

Zeppa et al (2004) reported 1-octen-3-ol to be a volatile marker only present in very ripe (stage III) *T. borchii* fruiting bodies (stages III = 71-100% mature; stage II = 31-70% mature; stage I = 6-30% mature; stage 0 = 0-5% mature). Out of 16 *T. borchii* samples analyzed in this study it is noteworthy that 1-octen-3-ol could also be detected at maturity stage II. Furthermore the same volatile was also missing in some fruiting bodies at maturity stage III. Taken together this suggests that maturity is not the major factor controlling 1-octen-3-ol biosynthesis.
